# Maxillary Osteomyelitis: A Rare Entity

**DOI:** 10.1155/2016/9723806

**Published:** 2016-08-25

**Authors:** Ayaaz Habib, Nagaraj Sivaji, Tauseef Ashraf

**Affiliations:** ^1^Pilgrim Hospital, Boston, Lincolnshire PE21 9QS, UK; ^2^Department of Otolaryngology, Pilgrim Hospital, Boston, Lincolnshire PE21 9QS, UK; ^3^Department of Radiology, Pilgrim Hospital, Boston, Lincolnshire PE21 9QS, UK

## Abstract

Osteomyelitis of the maxilla is now a rare event with the advent of antibiotics. The two predominant causes are odontogenic infections and sinusitis. Immunocompromised states such as diabetes, HIV, and malnutrition increase the risk of osteomyelitis. It is important to recognize this early as it is a difficult entity to treat with potentially serious consequences. We report an unusual case of right sided maxillary osteomyelitis in a lady with poorly controlled diabetes in rural Lincolnshire. Biopsy of the right maxillary bone showed features of acute osteomyelitis. This responded well to a prolonged course of oral antibiotics.

## 1. Introduction

Osteomyelitis is inflammation of the bone which begins as an infection of the medullary cavity with rapid involvement of the haversian systems and extension to the periosteum [1]. Osteomyelitis was a common disease before the advent of antibiotics. Today, osteomyelitis of the facial skeleton is a rare condition. It tends to occur more commonly in the mandible than in the maxilla as the maxilla has a significant collateral blood flow, thin cortical bones, and bone marrow with struts which make it less prone to infection [2].

Maxillary osteomyelitis can be classified based on the following causes: traumatic, rhinogenic, and odontogenic [3]. Factors which contribute to osteomyelitis are systemic diseases which compromise the immune system of an individual such as diabetes mellitus, HIV, malnutrition, and use of chemotherapeutic agents [4]. We hereby report a case of maxillary osteomyelitis in a lady who had recurrent maxillary sinusitis with poorly controlled diabetes mellitus.

## 2. Case Report

A 75-year-old lady presented to our ENT department complaining of pain and swelling in the right cheek for 3 months. She had a past medical history of recurrent maxillary sinusitis, chronic kidney disease stage 3, insulin dependent diabetes mellitus, ischaemic heart disease, asthma, and a previous cardiac arrest. On examination, there was swelling and erythema in the right maxillary region. There was no diplopia, nasal symptoms, or epistaxis. Her cranial nerve examination was unremarkable with no lymphadenopathy. Her throat and nasal examination was normal. Nasal endoscopy revealed a large antral opening with a crusty inside.

**Figure 1:**
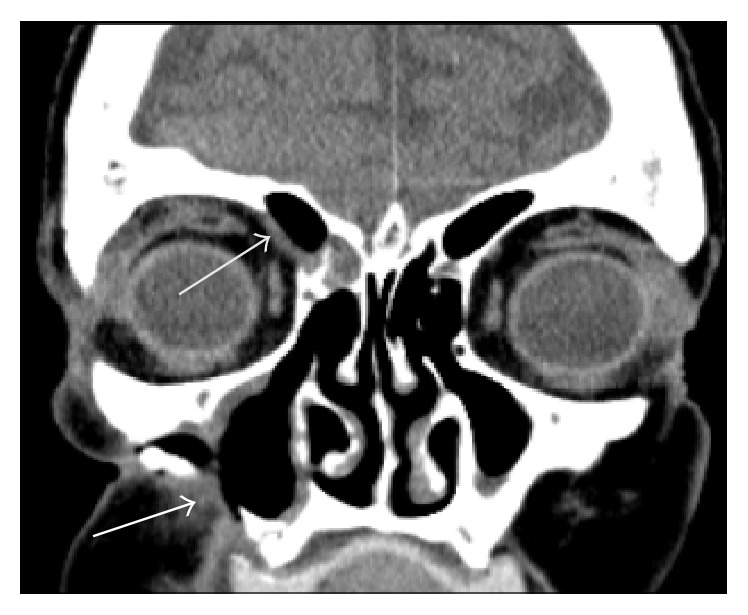
Coronal section CT image showing bony destruction in the lateral wall of the maxillary antrum with bone appearing to show some erosion and thickening. Bony dehiscence seen on the right superior orbital plate (white arrows) (courtesy of Dr. Tauseef Ashraf, Department of Radiology, Pilgrim Hospital, United Lincolnshire Hospitals NHS Trust).

**Figure 2:**
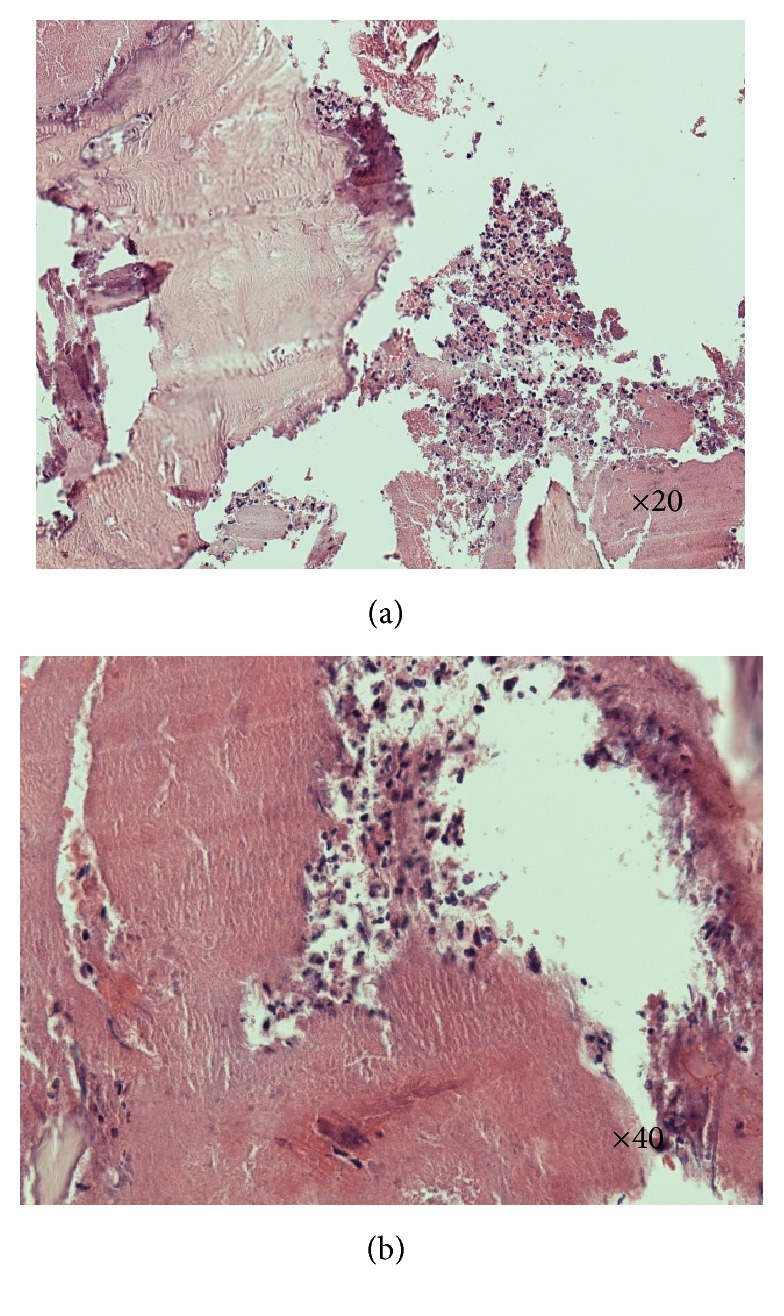
(a, b) H&E section showing necrotic bone and acute inflammation (courtesy of Dr. David Clark, Department of Pathology, Lincoln County Hospital, United Lincolnshire Hospitals NHS Trust).

A CT scan was performed (Figure 1) which showed bony destruction in the lateral wall of the right maxillary antrum with appearance of bone erosion and thickening. She was listed for examination of the nose and biopsy of the right maxillary sinus and antrum. Histopathology of the right maxillary sinus (Figures 2(a) and 2(b)) showed superficial piece of nonkeratinising squamous epithelium with underlying fibrous stroma showing acute inflammation.

There was evidence of necrotic bone showing marked acute inflammation consistent with osteomyelitis. The antral biopsy revealed patchy acute and chronic inflammation. Special stain for fungal organisms was negative. Given a high operative risk, we treated her with oral antibiotics alone with a good response. She is under regular follow-up.

## 3. Discussion

Osteomyelitis of the maxilla is a rare entity with the widespread use of antibiotics, early diagnosis, and intervention guided by new imaging modalities [5–7]. It has been reported extensively in literature, primarily in the form of case reports [4, 8]. It is important to consider the diagnosis in immunocompromised patients as it remains one of the most difficult to treat infectious diseases. In the past, osteomyelitis was encountered frequently and dreaded given its prolonged course, uncertainty of outcome, and possible disfigurement resulting from loss of teeth and bone [8]. Factors predisposing to osteomyelitis of the maxilla include dental infections, maxillary sinusitis, trauma, and radiation. The two main causes are dental infections and sinusitis [4]. When caused by sinusitis, it more frequently involves the frontal bone and rarely the maxilla due to its relatively well developed vascular supply and thin bone structure [9]. In this case, the main risk factor was poorly controlled diabetes mellitus and the patient had recurrent maxillary sinusitis which eventually progressed to involve the maxillary bone. According to Peravali et al., 68% of cases of maxillary osteomyelitis are related to diabetes mellitus as hyperglycaemia weakens the immune system by altering the blood flow distribution to the maxilla [4].

The treatments for maxillary osteomyelitis range from a noninvasive approach to a more invasive radical treatment [10]. A combination of antibiotic treatment with surgery has shown to be effective in treating the condition. Surgical treatment involves removal of loose teeth and sequestra, debridement, decortication, resection, and reconstruction [8]. In our case, the patient was treated with a prolonged course of amoxicillin and clavulanic acid alone making a good recovery.

## 4. Conclusion

It is important to consider osteomyelitis in immunosuppressed individuals as it is a difficult entity to treat. It may progress to involve infection of the cranial cavity and brain. It is imperative to suspect the diagnosis early and offer treatment with antibiotics. Optimal glycaemic control in diabetics is mandatory to prevent such infections.
